# Dysregulated PDGFR alpha expression and novel somatic mutations in colorectal cancer: association to RAS wild type status and tumor size

**DOI:** 10.1186/s12967-020-02614-3

**Published:** 2020-11-19

**Authors:** Nadia Ben Jemii, Haifa Tounsi-Kettiti, Hamza Yaiche, Najla Mezghanni, Amira Jaballah Gabteni, Emna Fehri, Chayma Ben Fayala, Sonia Abdelhak, Samir Boubaker

**Affiliations:** 1 Laboratory of Human and Experimental Pathology, Faculty of Science of Tunis, Institut Pasteur de Tunis, University Tunis El Manar, Tunis, Tunisia; 2 Laboratory of Human and Experimental Pathology, Faculty of Medicine of Tunis, Institut Pasteur de Tunis, University Tunis El Manar, Tunis, Tunisia; 3 Laboratory of Biomedical Genomics and Oncogenetics, Institut Pasteur de Tunis, University Tunis El Manar, Tunis, Tunisia

**Keywords:** Platelet derived growth factor receptor alpha, Colorectal cancer, RAS status, Mutations exon 18, Immunohistochemistry

## Abstract

**Background:**

Platelet derived growth factor receptor alpha (PDGFRα) has been considered as a relevant factor in tumor proliferation, angiogenesis and metastatic dissemination. It was a target of tyrosine kinase (TK) inhibitors emerged in the therapy of diverse cancers. In colorectal cancer, the commonly used therapy is anti-epithelial growth factor receptor (EGFR). However, both RAS mutated and a subgroup of RAS wild type patients resist to such therapy. The aim of this study is to investigate PDGFRα protein expression and mutational status in colorectal adenocarcinoma and their association with clinicopathological features and molecular RAS status to provide useful information for the identification of an effective biomarker that might be implicated in prognosis and treatment prediction.

**Methods:**

Our study enrolled 103 formalin fixed paraffin-embedded (FFPE) colorectal adenocarcinoma. PDGFRα expression was investigated by immunohistochemistry (IHC). Hotspot exon 18 of *PDGFRA* was studied by PCR followed by Sanger sequencing and RAS status was determined by real time quantitative PCR. Thirteen normal colon tissues were used as negative controls.

**Results:**

PDGFRα staining was detected in the cytoplasm of all tissues. Low expression was observed in all normal colon mucosa. In adenocarcinoma, 45% (45/100) of cases showed PDGFRα overexpression. This overexpression was significantly associated with mutations in exon 18 (P = 0.024), RAS wild type status (P < 10^–3^), tumor diameter (P = 0.048), whereas there was no association with tumor side (P = 0.13) and other clinicopathological features.

**Conclusion:**

Overexpression of PDGFRα in adenocarcinoma suggests its potential role in tumor cells growth and invasion. The association between PDGFRα overexpression in both tumor and stromal adenocarcinoma cells with RAS wild type status suggests its potential role in anti-EGFR therapy resistance and the relevance of using it as specific or adjuvant therapeutic target.

## Background

Prognosis and treatment of the heterogeneous disease, colorectal cancer (CRC), is challenging. CRC is the third leading cause of death in the world as well as in Tunisia with an age-standardized incidence and mortality rates of respectively 19.7 and 8.9 in the world and of 11.9 and 6.6 in Tunisia per 100,000 people (https://gco.iarc.fr/today/home). The evaluation of the prognosis and the response to therapy in CRC is based on several factors including TNM stage, some histopathological criteria and molecular testing for Rat sarcoma (RAS) mutation to select patients for anti-EGFR targeted therapy. Currently there is an increasing concern that these factors are limited in their ability to reflect the diversity of clinical behavior of colorectal cancer and the response to targeted therapy. Hence, they are not sufficient to discriminate patients with different molecular pathological profiles.

Consensus molecular subtypes (CMS) classification and other new molecular biomarkers are studied to assess the diagnosis and the prognosis of CRC and other malignancies as methaderin and octamer-binding transcription factor 4 (Oct4), but still not recommended for patients management [1, 2].

Platelet derived growth factor (PDGF) signaling pathway promotes processes of cancer aggressiveness as epithelial mesenchymal transition (EMT), tumor proliferation, growth and progression, angiogenesis, inhibition of apoptosis, recurrence and metastatic dissemination via the activation of various signaling pathway as PI3K/AKT and RAS/MAPK signaling pathways [3–5]. Platelet-derived growth factor receptors (PDGFR) and their ligands were reported as highly expressed in consensus molecular subtypes 4 (CMS4) colon tumors and identified as potential therapeutic targets for this subtype [6]. Dysregulation of PDGFR alpha (PDGFRα), one of receptors tyrosine kinase (RTK), has been reported in a broad range of cancer including glioblastoma, breast cancer, hepatocellular carcinomas, pancreatic cancer, and ovarian cancer [4, 7–9], either by protein overexpression or by the effect of mutations and chromosomal rearrangements. Moreover, this receptor has been approved by the Food and Drug Administration as therapeutic target for the treatment of patients with gastrointestinal stromal tumors (GISTs) (https://www.brimr.org/PKI/PKIs.htm). It was shown that Imatinib (PDGFRα inhibitor) can reduce the aggressive phenotype of CMS4 class colorectal tumors [6, 10].

Anti-epidermal growth factor receptor (EGFR) monoclonal antibody was the therapy commonly used for metastatic colorectal cancer patients with wild-type RAS (*KRAS*/*NRAS*) genes. Nevertheless, 25% of patients with RAS wild type (WT) status didn’t respond to this therapy [11]. Resistance could be explained by genetic alterations in other ancillary axes signaling pathways governing tumor growth, in addition to the tyrosine kinase receptor EGFR, representing a cross-RTK signaling switching that cannot be captured by targeting single RTK [12]. Recent data have demonstrated that EGF stimulates EGFR-PDGFRα transactivation and heterodimerization [13]. PDGFRα showed a crucial role in therapy resistance given its impact in both stromal and tumor cells which intensify tumor proliferation. In this context, genetic variabilities were identified in *PDGFRA* gene as associated to resistance toward anti-EGFR targeted therapy but the results still controversial [14, 15]. Moreover, the EGFR and PDGFR signaling pathways share large downstream signaling pathways as the activation of RAS genes. As a result, molecular RAS status could influence the expression level or interferes also with TK inhibition of other RTK than EGFR, including the PDGFα receptor.

This work aimed to explore the PDGFRα expression/mutational hot spot exon 18 status and its association with clinicopathological features and RAS status in colorectal adenocarcinoma in order to assess its potential role in prognosis and treatment prediction.

## Materials and methods

### Patients and tissue samples

A total of 116 formalin fixed and paraffin embedded (FFPE) tissues including 103 colorectal adenocarcinoma, and 13 normal colon mucosa as negative controls were collected from the archived tissues in Department of Human and Experimental pathology at Institut Pasteur de Tunis. Histological reports including tumor location, histological gradation and TNM status were collected.

### Pretreatment of formalin fixed and paraffin-embedded samples

For each sample, six sections of 4 μm-thick were obtained, 3 sections for DNA extraction, 2 sections for histopathological study (the first and the last sections to check the presence of tumor cells) and 1 section for immunohistochemical study. After each specimen, blades were changed to minimize the risk of cross-contamination.

### Histopathological study

Samples were stained with hematoxylin–eosin (HE) and examined by a Pathologist to confirm the histopathological diagnosis and to assess the proportion of tumor cells.

### Immunohistochemistry

Sections were deparaffinized in toluene, rehydrated with ethanol, and immersed in citrate antigenic retrieval buffer during 20 min in 95 °C water-bath and then cooled at room temperature for 20 min. Endogenous peroxidase activity was subsequently blocked with 3% hydrogen peroxide in methanol followed by incubation with protein block for 30 min. The sections were incubated with the primary antibody: anti-PDGFRα antibody (1:100; Santa Cruz Biotechnology, Inc.) for 1 h at room temperature. After phosphate-buffered saline (PBS) washing, tissue sections were incubated with biotinylated secondary antibody during 30 min, followed by incubation with novolink polymer (Leica Microsystems, Newcastle Ltd.) for 30 min. The antibody complex was visualized by the chromogens 3-amino-9-ethylcarbazole (AEC) and sections were counterstained with Mayer’s hematoxylin. Prostate tissue from department of Human and Experimental pathology of Institut Pasteur de Tunis was used as a positive control for primary antibody. For negative controls, the anti-PDGFRα antibody was replaced by PBS.

### Evaluation of immunohistochemical data

Tumoral and stromal cells were scored by a pathologist using the immuno-reactive-score (IRS) system. IRS system is the product of the staining intensity and the proportion of the positive stained tumor cells in comparison with negative tumor cells. Only cytoplasmic and or membranous staining were considered. Labeling intensity was scored from 0 to 3 as follow; 0: absence of staining, 1: weak staining, 2: moderate staining and 3: strong staining. The percentage of positive stained tumor cells was graded as follows: 0 for less than 10% of positive tumor cells, 0.5 for 10–50%, and 1 for more than 50% of positive tumor cells. The final scores obtained were 0; 0.5; 1; 1.5; 2 and 3. According to this score, PDGFRα expression was classified into two categories: low expression (IRS = 0–0.5) and high expression (IRS = 1–3).

### Molecular analysis

- DNA extraction and quantification
  DNA extraction from paraffin blocks was performed using the Qiagen (QIA) amp DNA Mini Kit (Qiagen, Courtaboeuf, France) according to the manufacturer’s instructions. DNA concentrations and purities were determined using a NanoDrop 2000c spectrophotometer (Thermo Fisher Scientific, Wilmington, Delaware).
- RAS mutation analysis
  The *KRAS*/*NRAS* mutational analysis was performed by the LightMix kit (TibMolBiol) according to the manufacturer’s instructions. Briefly, 3 probes (CTRL No-Clamped Control, LOW Clamped Mutation Analysis and HIGH Clamped Mutation Analysis) were used to detect specific mutations in the codons 12–13 of the second (first transcribed) exon of the *KRAS* gene. Whereas, 6 probes (N12-13, N59-61, N117, N146, K117 and K146) were used to identify mutations in the codons 12–13 (exon 2), codons 59–61 (exon 3), codon 117 and 146 (exon 4) of *NRAS* gene as well as codons 117 and 146 (exon 4) of the *KRAS* gene. Reaction mix was then inserted into Roche Diagnostics Light-Cycler instrument 480 to detect specific mutations.
- Exon 18 *PDGFRA* PCR amplification
  According to the Catalogue of Somatic Mutations in Cancer (COSMIC) database, the exon 18 of the *PDGFRA* gene is a hotspot pathogenic mutation site. Polymerase chain reaction (PCR) was performed using specific primer pair (Forward: 5^′^ GATCAGCCAGTCTTGCAG 3′; Reverse: 5′ CTCTAGAAGCAACACCTGAC 3′) covering 79 base pair (bp) of the intron 17–18, the totality of the exon 18 (123 bp) and 77 bp of the intron 18–19 of *PDGFRA* gene. The design of primers was carried out using the software “primer designing tools” by accessing the website “https://www.ncbi.nlm.nih.gov/tools/primer-blast/”.
  Extracted DNA was subjected to a PCR with the following parameters: 15 min initial denaturation at 94 °C, followed by 35 amplification cycles of 45 s at 94 °C, 45 s at 58 °C and 45 s at 72 °C, and a final extension step of 10 min at 72 °C, using a thermal cycler (BIORAD T100TM Thermal cycler, Life science research). The PCR products were then subjected to electrophoresis in a 1.5% agarose gel with syber safe.
- Exon 18 *PDGFRA *sanger sequencing
  The PCR products were sequenced on an automated sequencer (ABI 3500; Applied Biosystems, Foster City, CA, USA), using a cycle sequencing reaction kit (Big Dye Terminator kit, Applied Biosystems). Data were analyzed using the BioEdit Sequence Alignment Editor Version 7.0.5.3.
- Prediction tools
  *PDGFRA* mutations were predicted with different computational tools (Mutation taster: https://www.mutationtaster.org/, Human Splicing Finder (HSF): https://umd.be/Redirect.html, Sorting Intolerant From Tolerant (SIFT): https://sift.bii.a-star.edu.sg/, Protein Variation Effect Analyzer (PROVEAN): https://provean.jcvi.org/protein_batch_submit.php?species=human, Catalogue of Somatic Mutations in Cancer (COSMIC): https://cancer.sanger.ac.uk/cosmic, Ensembl: https://www.ensembl.org/index.html, UMD predictor: https://umd-predictor.eu/analysis.php and ClinVar: https://www.ncbi.nlm.nih.gov/clinvar/ in order to estimate splice site effects, protein damage or clinical signification. An online web-server HOPE was used to analyze the effects of point mutations on protein hydrophobicity, chemical and physical properties, spatial structure and function (https://www.cmbi.ru.nl/hope/) [16]. Moreover, effects of synonymous mutations on messenger ribonucleic acid (mRNA) folding were predicted using Mfold web server (https://www.bioinfo.rpi.edu/applications/mfold) [17]. The full mRNA sequence of *PDGFRA *(reference and mutated) and 123pb nucleotide sequence surrounding the synonymous variation were analyzed. To predict the amount of structural SNP, differences in single-strandedness count (ss-counts) (number of times each nucleotide is single stranded in a group of predicted foldings) were analyzed for each synonymous variation relative to the reference sequence in both full and partial sequence [18]. The most stable structure (having the lowest Gibbs free energy (ΔG) was used for analysis.

### Statistical analysis

IBM SPSS Statistics version 26.0 was used for all statistical analysis. Fisher’s exact test or χ^2^ test was performed to analyze association between PDGFRα expression, clinicopathological parameters, RAS status and molecular *PDGFRA* status. P < 0.05 was considered as statistically significant and P < 0.001 highly significant in all statistical analyses.

## Results

### Samples features

One hundred and three adenocarcinomas and 13 normal colon tissues were analyzed. The age of patients ranged from 22 to 88 years, with an average age of 57.4 years (SD, 13.2). There was a slight male predominance (63 male and 40 female) with a sex ratio equal to 1.5. Most of the adenocarcinomas were at stage pT3 (54/99 cases (54.5%) and left side located (49/98 cases (50‬ %). Histological study showed that the moderately differentiated adenocarcinoma was the most frequent histological subtype (64.1%). Table 1 summarizes clinicopathological data of the study series.

**Table 1:** Summary of clinicopathological features of the study group

Tissue samples	*N* (%)
Total number	103
Gender	
Female	40 (38.8%)
Male	63 (61.2%)
Location	
Colon	
Right-side	29 (29.6%)
Left-side	49 (50%)
Rectum	20 (20.4%)
Total	98
Diameter of tumor	
≤ 5 cm	54 (60.7%)
> 5 cm	35 (39.3%)
Total	89
Invasion of tumor	
T1	1 (1%)
T2	7 (7.1%)
T3	54 (54.5%)
T4	37 (37.4%)
Total	99
Lymph node metastasis	
N0	35 (35.4%)
N1	34 (34.3%)
N2	30 (30.3%)
Total	99
Histological grade	
Well differentiated	25 (24.3%)
Moderately differentiated	66 (64.1%)
Poorly differentiated	12 (11.7%)
Total	103

### Immunohistochemical PDGFRα expression

**Fig. 1:**
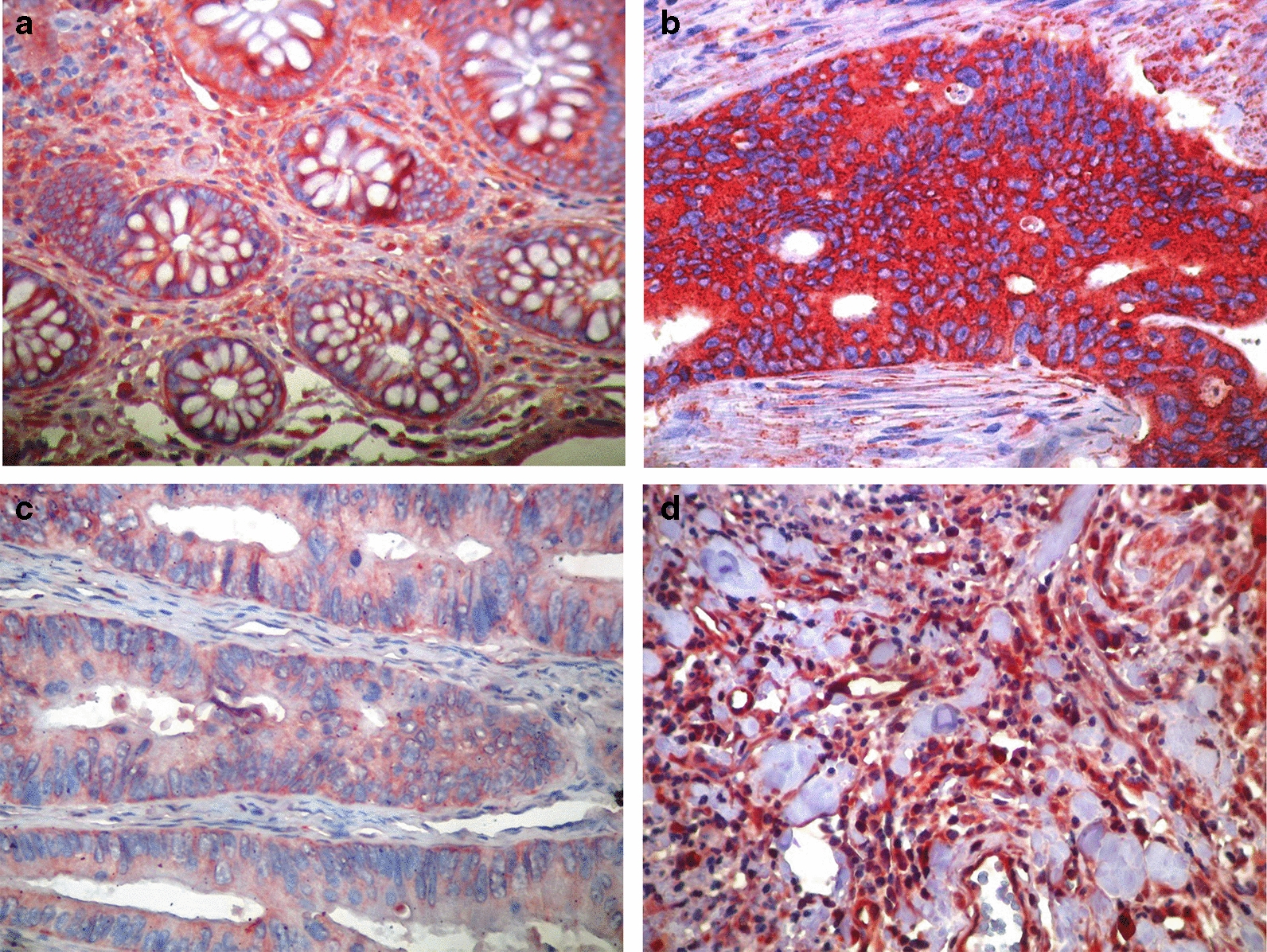
PDGFRα immunohistochemical expression pattern. **a** Weak staining of PDGFRα in normal colon epithelium. **b** Strong staining of PDGFRα in wild type RAS adenocarcinoma. **c** Weak staining of PDGFRα in mutated RAS adenocarcinoma. **d** Diffuse immunostaining in stromal cells (**a**–**c** magnification × 200; **d** magnification × 400)

The staining of PDGFRα was found in epithelial, endothelial and stromal (mononuclear elements of the stroma) cells. All normal colon mucosa showed low (IRS = 0–0.5) PDGFRα cytoplasmic staining strengthened by membranous immunolabelling (Fig. 1). In adenocarcinoma, 3 cases were eliminated because of nuclear staining. Among the remaining 100 samples, PDGFRα epithelial overexpression (IRS = 1–3) was found in 45% (45/100) and low expression in 55% (55/100) (Fig. 1). The expression pattern in ADK showed cytoplasmic staining in all cases, among them, 2 samples (0.02%) showed membranous and cytoplasmic labelling. These labellings were observed in low expression cases. PDGFRα overexpression was significantly associated to adenocarcinoma compared to normal tissues (P = 0.001). Focal to diffuse immunostaining of immune infiltrate and vessels was shown in the tumor microenvironment (Fig. 1). In adenocarcinoma stromal cells, 45% (45/100) of cases showed PDGFRα overexpression, 52% (52/100 cases) showed a low expression and 3% (3/100 cases) showed an absence of PDGFRα expression. Significant association was observed between PDGFRα overexpression in epithelial and stromal adenocarcinoma cells (P < 10^–3^).

With regards to clinicopathological features, PDGFRα overexpression observed in 45% of epithelial colorectal ADK was significantly associated with tumor diameter ≤ 5 cm (P = 0.048). No association was found between PDGFRα overexpression and other clinicopathological factors as shown in Table 2.

**Table 2:** Association between PDGFRα expression, clinicopathological parameters and RAS mutational status

Tissue samples	PDGFRα expression		P value
	Low (n = 55)	High (n = 45)	
Age (years)			0.447
< 50	13 (59.1%)	9 (40.9%)	
≥ 50	37 (54.4%)	31 (45.6%)	
Missing	5	5	
Gender			0.104
Male	30 (49.2%)	31 (50.8%)	
Female	25 (64.1%)	14 (35.9%)	
Missing	0	0	
Location			0.13
Colon	43 (57.3%)	32 (42.7%)	
Rectum	8 (40%)	12 (60%)	
Missing	4	1	
Diameter of tumor (cm)			0.048
≤ 5	23 (45.1%)	28 (54.9%)	
> 5	23 (65.7%)	12 (34.3%)	
Missing	9	5	
Invasion of tumor			0.644
T1	1 (100%)	0 (0%)	
T2	3 (50%)	3 (50%)	
T3	30 (56.6%)	23 (43.4%)	
T4	17 (47.2%)	19 (52.8%)	
Missing	4	0	
Lymph node metastasis			0.54
N0	20 (60.6%)	13 (39.4%)	
N1	18 (54.5%)	15 (45.5%)	
N2	14 (46.7%)	16 (53.3%)	
Missing	3	1	
Histological gradation			0.068
Well differentiated	18 (72%)	7 (28%)	
Moderately differentiated	33 (52.4%)	30 (47.6%)	
Poorly differentiated	4 (33.3%)	8 (66.7%)	
Missing	0	0	
RAS status			0.000
Wild type	17 (35.4%)	31 (64.6%)	
Mutated	38 (73.1%)	14 (26.9%)	
Missing	0	0	

### Mutational *RAS*/*PDGFRA *analysis

CRC patients were examined for molecular RAS (*KRAS*/*NRAS*) status resulting in 47.5% (49/103) with RAS wild type (WT) status and 52.4‬% (54/103) with mutated RAS status. The mutations were found in *KRAS* exon 2 gene in 44.6% (46/103) and in *KRAS *exon 3, 4; *NRAS* exons 2, 3, 4 gene in 7.7% (8/103). RAS WT status was highly associated to PDGFRα overexpression (P < 10^−3^) (Table 2).

The mutational analysis of the exon 18 of *PDGFRA* gene was done in 55 ADK and 3 normal samples. It revealed the presence of 18 variants, 5 in the intron 17–18, 10 in the exon 18 and 3 in the intron 18–19. Variant IVS17-50insA insertion (rs3830355) in intron 17–18 was found in all normal samples and in 53/55 ADK. All variations detected in the exon 18 and in the part of the intron 18–19 were absent in normal colon tissues. Among 10 mutations observed in the exon 18, 4 were non-synonymous and 6 were not reported previously. The Table 3 summarizes the characteristics of the different variations using different prediction tools.

**Table 3:** In silico analysis of PDGFRA variations in 55 CRC

Localisation	Sequence variation (nucleotide)	Variant Id dbSNP/Cosmic	Variant type	Protein variation	Frequency			Prediction of variant effect			ClinVar	Phenotype data (ensembl)
								Mutation taster/human splicing finder	PROVEAN/	UMD predictor		
									SIFT/			
					MAF	GenomAD	In our cohort (N = 55 CRC/%)		COSMIC (FATHMM)			
Reported variations												
Intronic (17–18)	c.2440-50_2440-49insA	rs3830355	Insertion	NA	0.49	0.805	53 (96.3%)	Polymorphism/	NA	NA	NA	NA
								Alteration of WT Branch Point + creation of an intronic ESE site				
Intronic (17–18)	c.2440-42C > T	rs1218113433	SNV	NA	< 0.01	3.9932e−06	20 (36.3%)	Polymorphism/	NA	NA	NA	NA
								No significant splicing motif alteration detected				
Intronic (18–19)	c.2562 + 28 G > A	rs767821722	SNV	NA	< 0.01	7.96476e−06	1 (1.8%)	Polymorphism/	NA	NA	NA	NA
								Alteration of an intronic ESS site + Creation of an intronic ESE site (probably no impact on splicing)				
Intronic (18–19)	c.2562 + 51C > T	rs1242272513	SNV	NA	< 0.01	6.97632e−06	1 (1.8%)	Polymorphism:Protein features might be affected + splice site changes/	NA	NA	NA	NA
								No significant splicing motif alteration detected				
Exonic	c.2464C > T	COSM5772696/COSV57271399	Non synonymous Somatic SNV	R822C	NA	NA	3 (5.4%)	Disease causing:amino acid sequence changed + protein features might be affected + splice site changes/	Deleterious/	Pathogenic	NA	Breast Tumor
									Damaging/			
								No significant splicing motif alteration detected	Pathogenic			
Exonic	c.2464C > A	COSM19324/ COSV57277919	Non synonymous Somatic SNV	R822S	NA	NA	2 (3.6%)	Disease causing: amino acid sequence changed + protein features might be affected + splice site changes/	Deleterious/	Pathogenic	NA	Soft tissue tumor
								Activation of an exonic cryptic acceptor site, with presence of one or more cryptic branch point(s)	Damaging/			
									Pathogenic			
Exonic	c.2472C > T	rs2228230/ COSM22413	Synonymous somatic SNV	V824V	0.45	0.183	10 (18.1%)	Polymorphism: protein features might be affected + splice site changes/	Neutral/	Polymorphism	Benign	Gastrointestinal stromal tumors, Idiopathic hypereosinophilic syndrome
								Creation of an exonic ESS site	Tolerated/			
									Pathogenic			
Exonic	c.2517G > T	rs1213039385/COSM6100284	Synonymous somatic SNV	L839L	< 0.01	3.97842e-06	1 (1.8%)	Disease causing: protein features might be affected + splice site changes/	Neutral/	Polymorphism	NA	Lung tumor
									Tolerated/			
								Creation of an exonic ESS site + Alteration of an exonic ESE site	Pathogenic			
Unreported variations												
Intronic (17–18)	g.56700C > T	NA	SNV	NA	NA	NA	26 (47.2%)	Polymorphism: protein features might be affected + splice site changes/	NA	NA	NA	NA
								No significant splicing motif alteration detected				
Intronic (17–18)	g.56701 T > G	NA	SNV	NA	NA	NA	4 (7.2%)	Polymorphism: protein features might be affected + splice site changes/	NA	NA	NA	NA
								alteration of an intronic ESS site + Creation of an intronic ESE site (probably no impact on splicing)				
Intronic (17–18)	g.56717C > G	NA	SNV	NA	NA	NA	5 (9%)	Polymorphism/	NA	NA	NA	NA
								Alteration of WT branch point				
Exonic	c.2514C > T	NA	Synonymous SNV	G838G	NA	NA	3 (5.4%)	Disease causing/	Neutral/	Probably pathogenic	NA	NA
								Activation of an exonic cryptic donor site + Creation of an exonic ESE site	Tolerated/			
									NA			
Exonic	c.2481A > T	NA	Synonymous SNV	A827A	NA	NA	5 (9%)	Disease causing: protein features might be affected + splice site changes/	Neutral/	Polymorphism	NA	NA
									Tolerated/			
								No significant splicing motif alteration detected	NA			
Exonic	c.2459C > T	NA	Non synonymous SNV	A820V	NA	NA	5 (9%)	Disease causing: amino acid sequence changed + protein features might be affected + splice site changes/	Deleterious/	Pathogenic	NA	NA
								Activation of an exonic cryptic donor site + Creation of an exonic ESS site + Alteration of an exonic ESE site	Damaging/			
									NA			
Exonic	c.2496G > A	NA	Synonymous SNV	V832V	NA	NA	2 (3.6%)	Disease causing: protein features might be affected + splice site changes/	Neutral/	Polymorphism	NA	NA
								Creation of an exonic ESS site + Alteration of an exonic ESE site	Tolerated/			
									NA			
Exonic	c.2520C > A	NA	Synonymous SNV	A840A	NA	NA	1 (1.8%)	Disease causing: protein features might be affected + splice site changes/	Neutral/	Polymorphism	NA	NA
								Creation of an exonic ESS site + Alteration of an exonic ESE site	Tolerated/			
									NA			
Exonic	c.2507A > T	NA	Non synonymous SNV	D836V	NA	NA	1 (1.8%)	Disease causing: protein features might be affected + splice site changes/	Deleterious/	Pathogenic	NA	NA
								Alteration of an exonic ESE site	Damaging/			
									NA			
Intronic (18–19)	g.56935G > A	NA	SNV	NA	NA	NA	2 (3.6%)	Polymorphism/	NA	NA	NA	NA
								Alteration of an intronic ESS site + Creation of an intronic ESE site (probably no impact on splicing)				

*dbSNP* single nucleotide polymorphism database, *SNV* single nucleotide variant, *MAF* minor allele frequency, *NA* Results not available with that bioinformatic tool

Mutations in exon 18 were significantly associated only with PDGFRα overexpression (P = 0.024) and mutations in the intron 18–19 were significantly associated with well differentiated adenocarcinoma (P = 0.035) (Table 4). There was no association otherwise between different variations in *PDGFRA* gene and clinicopathological characteristics (Table 4).

**Table 4:** Association between PDGFRA mutational status and clinicopathological parameters, PDGFRα expression and RAS mutational status

Tissue samples (N = 55 CRC)	*PDGFRA* intron 17–18		*PDGFRA* exon 18		*PDGFRA* intron 18–19	
	Present	P-value	Present	P-value	Present	P-value
Age (years)		0.239		0.5		0.643
< 50 years	7 (70%)		4 (40%)		0 (0%)	
≥ 50 years	21 (51.2%)		19 (46.3%)		2 (4.9%)	
Gender		0.43		0.55		0.599
Male	17 (48.6%)		15 (42.9%)		1 (2.9%)	
Female	11 (55%)		9 (45%)		1 (5%)	
Location		0.119		0.083		0.339
Colon	25 (56.8%)		22 (50%)		1 (2.3%)	
Rectum	3 (30%)		2 (20%)		1 (10%)	
Diameter of tumor		0.521		0.258		0.635
≤ 5 cm	16 (51.6%)		15 (48.4%)		1 (3.2%)	
> 5 cm	11 (55%)		7 (35%)		1 (5%)	
Invasion of tumor		0.228		0.957		0.952
T2	2 (100%)		1 (50%)		0 (0%)	
T3	16 (55.2%)		13 (44.8%)		1 (3.4%)	
T4	10 (41.7%)		10 (41.7%)		1 (4.2%)	
Lymph node metastasis		0.077		0.924		0.125
N0	14 (70%)		9 (45%)		0 (0%)	
N1	6 (33.3%)		7 (38.9%)		2 (11.1%)	
N2	8 (50%)		7 (43.8%)		0 (0%)	
Histological gradation		0.283		0.871		0.035
Well differentiated	9 (69.2%)		5 (38.5%)		2 (15.4%)	
Moderately differentiated	16 (47.1%)		15 (44.1%)		0 (0%)	
Poorly differentiated	3 (37.5%)		4 (50%)		0 (0%	
PDGFRα expression		0.089		0.024		0.103
Low	16 (43.2%)		4 (22.2%)		2 (11.1%)	
High	12 (66.7%)		20 (54.1%)		0 (0%)	
RAS status		0.333		0.08		0.666
Wild type	15 (46.9%)		17 (53.1%)		1 (3.1%)	
Mutated	13 (56.5%)		7 (30.4%)		1 (4.3%)	

### Effect of the mutations on the protein structure and function

**Fig. 2:**
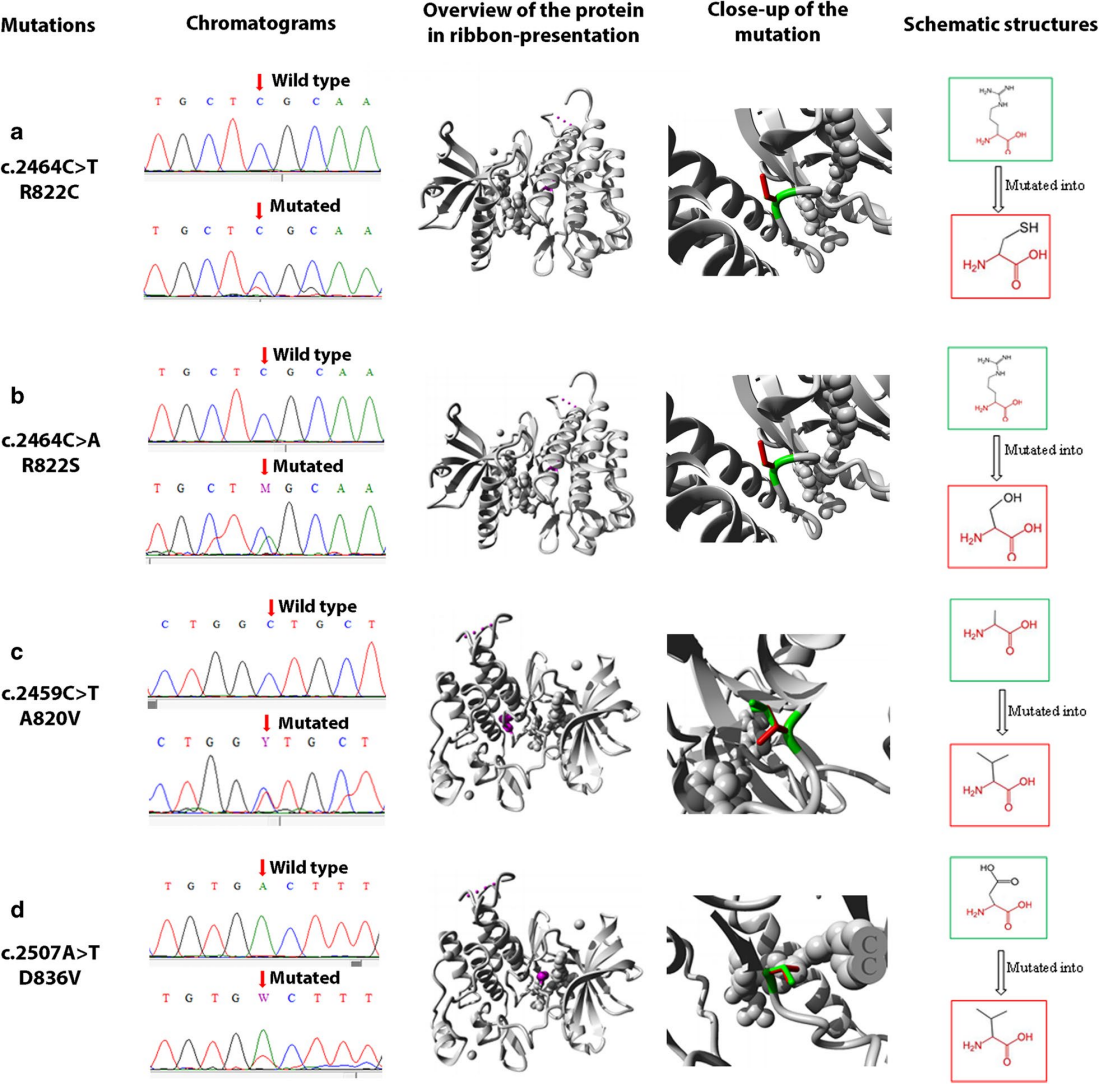
The effect of non-synonymous mutations on 3D PDGFRα structure. Sanger sequencing chromatograms of *PDGFRA* exon 18 show the wild and mutated sequence of non-synonymous variations (c.2464C > T, c.2464C > A, c.2459C > T and c.2507A > T). Overviews of protein in ribbon-presentation show the protein in grey and the side chain of the mutated residue in magenta (shown as small balls). The Close-up of mutations (seen from a slightly different angle) shows the protein in grey and the side chains of both the wild-type and the mutant residue in green and red respectively. Schematic structures show the original (green) and the mutant (red) amino acid. The backbone, which is the same for each amino acid, is colored red. The side chain, unique for each amino acid, is colored black

We found four non-synonymous mutations in the exon 18 (c.2464C > T, c.2464C > A, c.2459C > T, c.2507A > T) which spatial effect on protein domain are shown in Fig. 2. In fact, the c.2464C > T and c.2464C > A mutations change the Arginine at position 822 into a cysteine and a serine respectively. These mutations share the same properties: the mutant residue is smaller, has a neutral charge and is more hydrophobic than the wild-type residue. Their localization within a protein kinase domain will cause loss of hydrogen bonds in the core of the protein and as a result disturbs correct folding. Moreover, these mutations are located in an important domain for the protein activity which is in contact with other domains involved in binding or in protein activity. The interaction between these domains could be disturbed by these mutations, which might affect protein function or signal transduction. The mutation c.2459C > T change the alanine into a Valine at position 820. The mutant residue is bigger than the wild-type residue. This mutation, located within a domain tyrosine kinase, introduces an amino acid with different properties, which can disturb this domain and abolish its function. The c.2507A > T mutation changes an aspartic acid into a valine at position 836. This mutant residue is smaller, has a neutral charge and is more hydrophobic than the wild-type residue. The difference in properties between wild-type and mutation can easily disturb ionic, domains and ligand interaction which might affect protein function and structure.

**Fig. 3:**
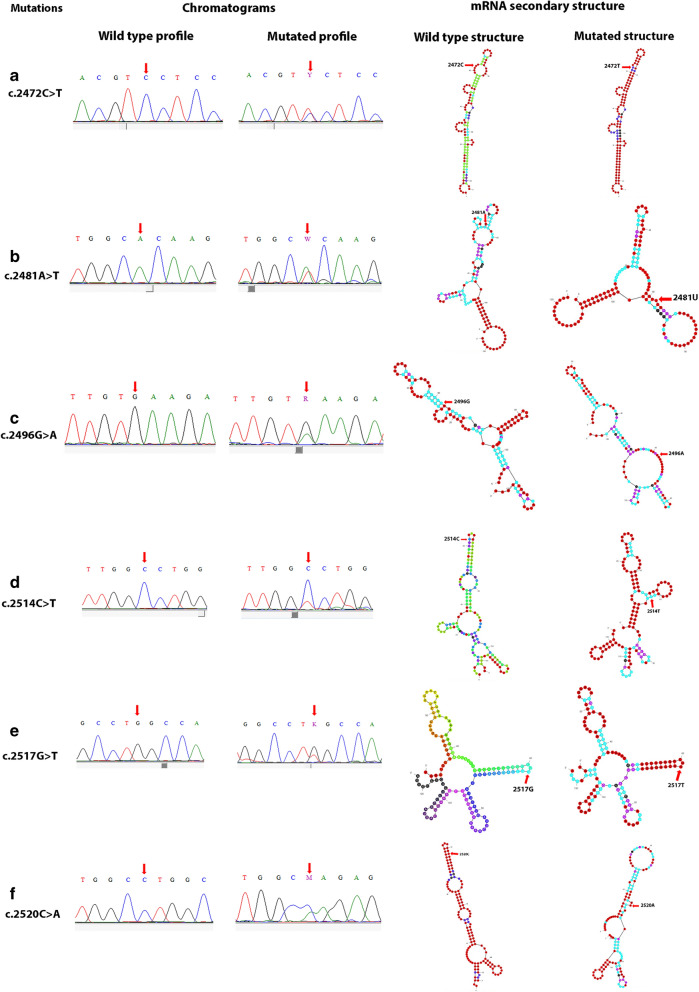
The effect of synonymous variations on mRNA secondary structure. Sanger sequencing chromatograms show the wild and mutated sequence of synonymous *PDGFRA* exon 18 variations (c.2472C > T, c.2481A > T, c.2496G > A, c.2514C > T, c.2517G > T and c.2520C > A). The effect of synonymous variations on partial mRNA (123pb) secondary structure is compared to the reference sequence at the same position (arrows)

Our results showed also the presence of 6 synonymous variations (c.2472C > T, c.2481A > T, c.2496G > A, c.2514C > T, c.2517G > T and c.2520C > A). Except the c.2481A > T, these variations were predicted to induce splicing site alteration by HSF tool (Table 3). In order to test if they change mRNA secondary structure, we used Mfold web server. All synonymous variations changed ss-count as compared to the full length and the partial reference sequence which might change the mRNA secondary structure except for the c.2517G > T (data not mentioned). Partial mRNA folding structure of mutant compared to their wild type sequence were shown in Fig. 3. The mRNA folding carrying c.2517G > T was similar to that of WT. Other synonymous variations may lead to the change of mRNA secondary structure.

## Discussion

To our knowledge, this is the first study investigating PDGFRα protein expression and molecular profiles in colorectal cancer and correlating these profiles with clinicopathological features and RAS status.

### PDGFRα staining pattern and mutational status in CRC

In this work, immunohistochemistry results showed an immunostaining chiefly in the cytoplasm except for 2 ADK having membranous joined by cytoplasmic staining. These 2 cases have a low PDGFRα expression. Only cytoplasmic stain was found by Wehler and al using the same anti**-**PDGFRα antibody in all colorectal cancer samples studied [4]. They suggest that the cytoplasmic localization is the result of an impaired ubiquitination mechanism; possibly due to alterations undoing indirect link between PDGFRα and c-Cbl required and sufficient for endocytosis and lysosomal degradation [4, 19]. PDGFRα cytoplasmic localisation might extend lifetime and/or execute specific functions as the activation of unconventional signaling pathways like STAT, c-Jun and PLCγ pathways [20–24].

In the present study, we found that PDGFRα was weakly expressed in all control cases which is in accordance with other studies [4, 15, 25–27].

In colorectal ADK, our results demonstrate the presence of PDFGRα in all cases which was in agreement with bibliographic data. In fact, Wehler et al. have found that PDGFRα was present in 82.8% (82/99 cases) of human colorectal cancer specimens [4]. In the same context, Schimanski et al. have found that PDGFRα was expressed in 84.9% (79/93 cases) of human colorectal cancers [15]. The analysis of our series demonstrated that 45% (45/100) of ADK cases showed PDGFRα overexpression which was significantly associated to ADK as compared to normal mucosa (P = 0.001). Dai et al. revealed by cDNA microarray analysis of 16 cases of CRC and proximal non-cancerous colorectal mucosa, the overexpression of PDGFRα in colorectal cancers as compared to that in normal tissues (ratio = 4.81 ± 0.14) [26]. The same result was showed by Li et al. using the western blot analysis of 176 colon cancer specimens and normal biopsies [27]. Overexpression proportion variations could be the consequence of the use of different methodology, IHC scoring, and different number of cases. In this study, we used 103 cases of CRC and 13 cases as normal controls. This could explain the proportion difference of high expression regarding to normal controls.

Besides expression of PDGFRα in tumor colorectal cells, we noted the presence of focal to diffuse PDGFRα immunostaining in various mesenchymal stromal cells including inflammatory cells and vessels. In ADK stromal cells, our results showed that PDGFRα was expressed in 97% (97/100 cases) of specimens. These were corroborated by previous results of Wehler et al. that showed PDGFRα expression in 70% of stromal colorectal carcinoma cells [4]. However, Bian et al. reported a moderately PDGFRα expression [25]. Our results showed an association between PDGFRα expression in tumor and stromal cells (P < 10^−3^, respectively). These results suggest that in ADK, stromal cells could intensify tumor growth. In fact, tumor-associated stroma formed by ostensibly normal cells was considered as active participants in tumorigenesis which leads to cancer progression and metastatic dissemination by interacting with cancer cells [28, 29].

In our study, 54.1% of samples with PDGFRα present mutation in exon hotspot18 which encodes the tyrosine kinase domain II, a highly conserved region in *PDGFRA* gene. We noticed that mutations in the flanking intron parts have no impact on PDGFRα expression given the absence of association between these mutations and the overexpression of PDGFRα. However, in the coding region, we have found 4 non synonymous mutations which changed 3D PDGFRα structure according to the HOPE web server (https://www.cmbi.ru.nl/hope/). Two non-synonymous mutations were previously reported: the c.2464C > T (COSM5772696) was described in colorectal cancer [14], as well as in other cancers [30] (COSMIC database: https://cancer.sanger.ac.uk/cosmic/gene/analysis?ln=PDGFRA), and, the c.2464C > A (COSM19324) identified only in gastrointestinal stromal tumor [31]. Our study also revealed the presence of 6 synonymous variations which might affect protein features, splicing sites or mRNA folding or stability according to mutation taster, HSF and mfold RNA prediction tools. Similar results were reported for other synonymous variation in CRC and other malignancy via the same mechanisms mentioned above [32–34]. This would have phenotypic effect on protein expression [35, 36]. Our analysis showed a non-significant but a tendency toward association between the presence of synonymous variations and the PDGFRα overexpression (P = 0.069; data not shown). However, it was shown by in vitro analysis that the synonymous polymorphism (c.2472C > T), found in our study, reduces PDGFRα expression in acral melanoma via decreasing its mRNA and protein stability and its downstream signaling activity (MAPK and PI3K/AKT) [37]. In the same study, this polymorphism was associated to better survival [37]. However, it was reported as significantly associated with worse prognosis in renal cell carcinoma [38]. Effect of synonymous variation on protein expression could be the result of organ specificity. In our study, synonymous and non-synonymous mutations observed in the coding region of the *PDGFRA* gene were not observed in normal colon tissues. Therefore, the presence of these mutations in the coding conserved region could explain that high PDGFRα expression (P = 0.024) might lead to colorectal carcinogenesis. In the same context, work on colorectal cancer has demonstrated the presence of other activating mutation as the D842V in the exon 18 in 2 of 322 ADK cases [39]. This mutation investigated for the determination of the response to Imatinib GIST therapy wasn’t detected in our study. In contrast to our results, Shao et al. showed the absence of *PDGFRA* mutations in 46 human colorectal cancer samples [40].

Moreover, our results showed that 45.9% (17/37) of cases with PDGFRα overexpression have no mutation in the exon 18. Overexpression of PDGFRα in these cases could be explained by the presence of mutations in other exons of the gene [14, 39] or other activating mechanisms including gene amplification, autocrine loop activation, chromosomal alterations producing PDGFRα fusion with other gene and the deregulation of miRNA as miR-34a [4, 24, 27]. Increased PDGFRα expression could be caused also by the activating effect of signaling pathway as the Sonic Hedgehog pathway [41].

### PDGFRα expression and clinicopathological parameters

In an attempt to explore the role of PDGFRα in neoplastic progression, we correlated its expression with clinicopathological parameters. Our study showed an association between high PDGFRα expression in ADK cells and tumor size ≤ 5 cm (P = 0.048). Such association was not previously reported in colorectal cancer. The role of tumor diameter in CRC prognosis and recurrence remains controversial. In fact, several studies identified large tumor diameter as risk factor for recurrence, postoperative complications after laparoscopic surgery of advanced rectal cancer, metastasis and poor prognosis [42–45]. However, other studies revealed that small tumor size was associated to higher recurrence, poor survival and prognostic features [46–48]. According to the previous reports, correlation between PDGFRα overexpression and progressed International Union against Cancer (UICC) stages III/IV and lymph node metastasis was reported in older patients with colorectal sporadic cancer suggesting its important role in colorectal cancer dissemination [4]. However, our data showed no significant association between PDGFRα overexpression and T status (P = 0.644), N status (P = 0.54), age class (P = 0.447) or histological gradation (P = 0.068). Furthermore, our results showed the absence of significant association (P = 0.083) between the presence of mutations in exon 18 of ADK cases and the colon location. Several features identify colon and rectal cancer like complications, treatment, short-term mortality, long-term survival and recurrences [49]. Gene expression profiles and activating signaling pathways also vary according to tumor location as MAPK signaling pathway which was downregulated in rectal cancer [50]. PDGFRα was reported as highly expressed in CMS4 colon tumors [6]. This molecular subtype is composed mainly of left-sided primary tumors and tended to be diagnosed at stage III and IV [51, 52]. As a result, our findings suggest that PDGFRα may have an effect on colorectal cancer prognosis. Larger samples could improve the significance of the associations.

### Association between PDGFRα protein expression and mutational *RAS* status

**Fig. 4:**
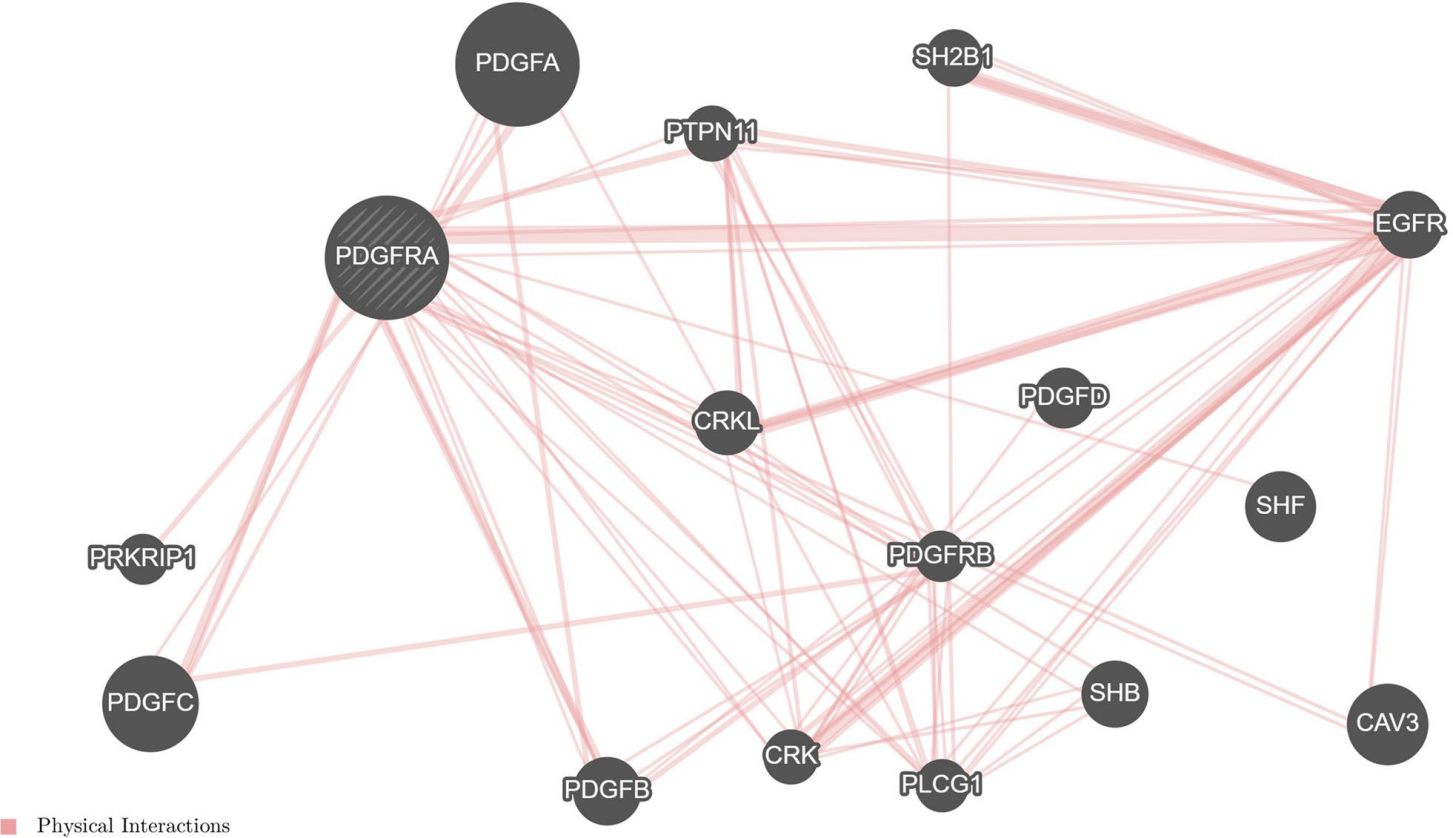
PDGFRA-EGFR interaction as analyzed by GeneMANIA prediction server (https://genemania.org/). *PRKRIP1:* PRKR interacting protein 1 (IL11 inducible), *PDGFA/B/C/D:* platelet derived growth factor subunit A/B/C/D, *PDGFRA/B:* platelet derived growth factor receptor A/B, *PTPN11:* tyrosine-protein phosphatase non-receptor type 11, *CRKL:* v-crk avian sarcoma virus CT10 oncogene homolog-like, *CRK:* CRK proto-oncogene, Adaptor Protein, *SH2B1:* Src homology 2 B adaptor protein 1, *PLCG1:* phospholipase C gamma 1, *SHB:* SH2 domain containing adaptor protein B, *SHF:* Src homology 2 domain containing F, *EGFR:* epidermal growth factor receptor, *CAV3:* caveolin 3

Our study showed that 64.5% (31/48 cases) of RAS WT ADK cases overexpressed PDGFRα (P < 10^−3^) possibly due to the presence of mutations in *PDGFRA* exon 18 (P = 0.08). These findings suggest that PDGFRα may represent a driver of tumor progression in RAS WT subgroup. In contrast to our results, Schimanski et al. have identified an association between PDGFRα expression and KRAS codon 12 or 13 mutation [15]. Currently, the use of EGFR targeted therapies in CRC is limited to patients with wild-type RAS genes. However, even with RAS WT status, resistance to this therapy occurs in 25% of patients [11]. Resistance could be explained by genetic alterations in other ancillary axes signaling other than EGFR pathway that cannot be captured by targeting single RTK [12]. Recent data have demonstrated that EGF stimulates EGFR-PDGFRA transactivation and heterodimerization [13]. The Fig. 4 generated by GeneMANIA bioinformatic analysis supported our hypothesis (https://genemania.org/). This figure showed EGFR-PDGFRA physical interaction collected from primary studies found in protein interaction databases, including BioGRID and Pathway Commons [53].

**Fig. 5:**
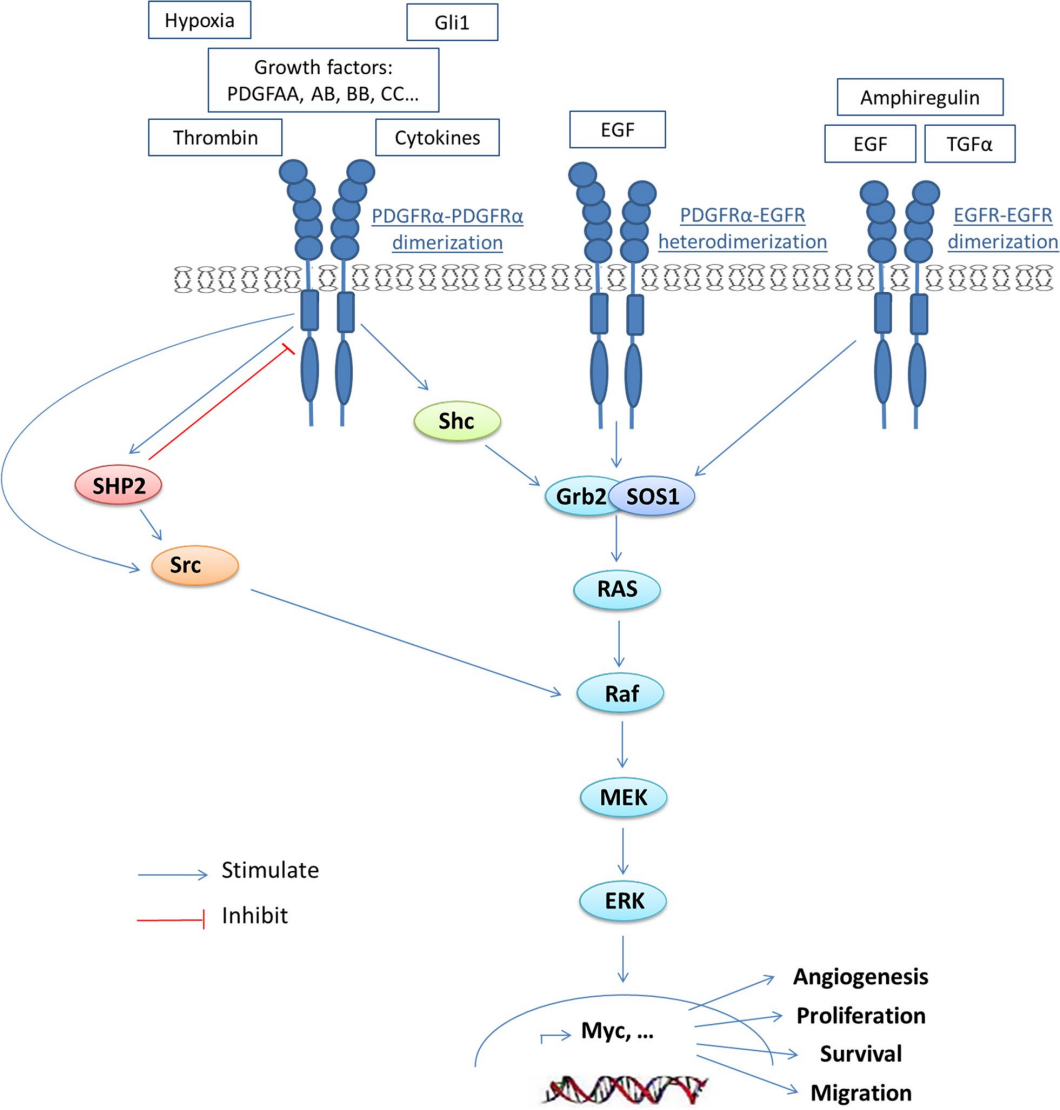
PDGFRα/EGFR signaling activation and effects on RAS/MAPK pathway. *SHP2* (*PTPN11*): Src Homology Region 2-Containing Protein Tyrosine Phosphatase-2; Src: Non-Receptor Tyrosine Kinase; Shc: Src Homology 2 Domain-Containing-Transforming Protein, *Grb2:* growth factor receptor-bound protein 2, *SOS1:* Son of sevenless homolog 1, *RAS:* Rat sarcoma, *RAF:* rapidly accelerated fibrosarcoma, *MEK:* mitogen-activated protein kinase kinase, *ERK:* extracellular signal-regulated kinases, *Gli1:* GLI Family Zinc Finger 1, *EGF:* epidermal growth factor, *TGFα:* transforming growth factor alpha, *PDGFAA/AB/BB/CC:* platelet derived growth factor AA/AB/BB/CC

Moreover, the EGFR and PDGFRα share large downstream signaling pathways as the activation of RAS/MAPK pathway via various proteins (Fig. 5).

These evidences suggest that protein overexpression or genetic variabilities identified in *PDGFRA* exon 18 could explain this resistance. By analyzing the *PDGFRA* exon 18 mutations, we have detected the c.2464C > T mutation in 3 colorectal ADK cases all of which have WT RAS status. The c.2464C > T and other mutations among exons (16, 22, 19, 4, 7, 15, 8 and 10) were reported in CRC patients with KRAS WT status resistant to anti-EGFR targeted therapy [14]. Li et al. reported that the absence of mutations in exon 18 and 15 of the *PDGFRA* gene and in other prognostic genes (KRAS, NRAS, BRAF and PIK3CA), showed a better response rate with anti-EGFR therapy (cetuximab) [39]. As a result, given the existence of different activating mutations in the *PDGFRA* gene and their high association with overexpression, immunohistochemistry could be used as a test to identify patients resistant toward anti-EGFR targeted therapy and prognostic prediction. It was demonstrated that the combination of PDGFR and EGFR inhibitors (imatinib versus cetuximab) in colorectal tumor graft with mutant *PDGFRA* R981H (exon 22), identified as a mechanism of primary resistance to EGFR blockade, has a strong anti-tumor activity but with a short-lived effect [14]. This non-significant combination could be the result of the existence of other mutations in the *PDGFRA* gene that cause resistance to imatinib therapy. Moreover, imatinib is a multi-receptors tyrosine kinase inhibitor so that it is not known to what degree their therapeutic effects are related to PDGFRα inhibition. As a result, it will be better to use specific neutralizing PDGFRα antibodies in combination with anti-EGFR therapy for patients with RAS WT status. Furthermore, our results showed that 26.9% (14/52 cases) of mutated RAS ADK cases overexpressed PDGFRα. Bevacizumab (anti vascular endothelial growth factor (VEGF) therapy) is prescribed for colorectal cancer with RAS mutated status. Despite improving progression-free survival, the survival benefit of Bevacizumab remains limited due to the acquired resistance [54]. It was found that VEGF-A directly binds to PDGFRα and induce their activations [55]. PDGFRα overexpression might be one of resistance mechanisms. These findings suggest that specific neutralizing PDGFRα antibodies in combination with anti-VEGF therapy could be used for patients with mutated RAS status.

In order to validate these results, larger samples (controls and CRC tissues) and follow of response to targeted therapy are needed.

## Conclusion

PDGFRα was significantly overexpressed in ADK compared to normal mucosa which may suggest its potential role in the development or the sustain of tumor cells. Furthermore, high PDGFRα expression was significantly associated to RAS WT status (P < 10^–3^) suggesting its role in the resistance to anti-EGFR and thus the possible inclusion of this protein in the panel of predictive biomarkers of response to anti-EGFR therapies. In another way, the fact that PDGFRα was expressed by tumor, surrounding stromal and endothelial cells, makes this receptor a good target by specific neutralizing antibodies. The cytoplasmic mislocalization of this receptor could confer to therapy a high degree of specificity. The IHC expression of PDGFRα could be a good option to select patients for associated anti PDGFR therapy. We should therefore study in more detail the genetic alteration of the whole *PDGFRA* gene which could be behind the alteration of its expression and its localization as well as determining the follow-up of patients with an overexpression of the PDGFRα protein and WT RAS status.

## Data Availability

All data generated or analyzed during this study are included in this published article.

## Abbreviations

CRC: Colorectal cancer
ADK: Adenocarcinoma
CMS: Consensus molecular subtypes
PDGF: Platelet derived growth factor
PDGFR: Platelet derived growth factor receptor
EMT: Epithelial mesenchymal transition
RTK: Receptor tyrosine kinase
GIST: Gastrointestinal stromal tumors
EGFR: Epithelial growth factor receptor
FFPE: Formalin fixed paraffin-embedded
DNA: Deoxyribonucleic acid
HE: Hematoxylin–eosin
IRS: Immuno-reactive-score
KRAS: V-Ki-RAS2 Kirsten rat sarcoma viral oncogene homolog
NRAS: Neuroblastoma RAS viral oncogene homolog
IHC: Immunohistochemistry
AEC: Chromogens 3-amino-9-ethylcarbazole
PBS: Phosphate-buffered saline
COSMIC: Catalogue of somatic mutations in cancer
HSF: Human splicing finder
SIFT: Sorting intolerant from tolerant
PROVEAN: Protein variation effect analyzer
mRNA: Messenger ribonucleic acid
ΔG: Gibbs free energy
SNV: Single nucleotide variant
MAF: Minor allele frequency
dbSNP: Single nucleotide polymorphism database
SNV: Single nucleotide variant
MAF: Minor allele frequency
ICC: Interstitial cells of Cajal
SMC: Smooth muscle cells
UICC: International union against cancer
BRAF: V-raf murine sarcoma viral oncogene homolog B1
PIK3CA: Phosphatidylinositol-4,5-bisphosphate 3-kinase catalytic subunit alpha
